# Composition, Seasonal Variation, and Biological Activities of *Lantana camara* Essential Oils from Côte d’Ivoire

**DOI:** 10.3390/molecules25102400

**Published:** 2020-05-21

**Authors:** Fatimata Nea, Didjour Albert Kambiré, Manon Genva, Evelyne Amenan Tanoh, Esse Leon Wognin, Henri Martin, Yves Brostaux, Félix Tomi, Georges C. Lognay, Zanahi Félix Tonzibo, Marie-Laure Fauconnier

**Affiliations:** 1 Laboratory of Biological Organic Chemistry, UFR-SSMT, University Félix Houphouët-Boigny, 01 BP 582 Abidjan 01, Ivory Coast; dakambire@gmail.com (D.A.K.); evelynetanoh5@gmail.com (E.A.T.); esse.wognin@yahoo.fr (E.L.W.); tonzibz@yahoo.fr (Z.F.T.); 2 Laboratory of Chemistry of Natural Molecules, Gembloux Agro-Bio Tech, University of Liège, Passage des Déportés 2, B-5030 Gembloux, Belgium; m.genva@uliege.be (M.G.); henri.martin@uliege.be (H.M.); marie-laure.fauconnier@uliege.be (M.-L.F.); 3 Laboratory of Instrumentation Image and Spectroscopy, National Polytechnic Institute Felix Houphouët-Boigny, BP 1093 Yamoussoukro, Ivory Coast; 4 Applied Statistics, Computer Science and Modelling Unit, Gembloux Agro-Bio Tech, University of Liège, avenue de la Faculté d’Agronomie 8, B-5030 Gembloux, Belgium; y.brostaux@uliege.be; 5 Université de Corse-CNRS, UMR 6134 SPE, Equipe Chimie et Biomasse, Route des Sanguinaires, F-20000 Ajaccio, France; tomi_f@univ-corse.fr; 6 Analytical Chemistry Laboratory, Gembloux Agro-Bio Tech, University of Liège, Passage des Déportés 2, B-5030 Gembloux, Belgium; georges.lognay@uliege.be

**Keywords:** *Lantana camara*, essential oil composition, thymol, vegetative period, antioxidant activity, anti-inflammatory activity, insecticidal activity

## Abstract

This work aims to study the variations in the composition of *Lantana camara* leaf, flower, and stem essential oils over two years. *L. camara* organs were harvested in Bregbo (East Côte d’Ivoire) each month from June 2015 to June 2017. The essential oils were obtained by hydrodistillation and characterized by GC-MS and ^13^C NMR. Eighty-four compounds accounting for 84.4–99.1% of the essential oils have been identified. The essential oils hydrodistillated from *L. camara* are dominated by sesquiterpenes such as (*E*)-β-caryophyllene and α-humulene, which were found in all samples. Some monoterpenes such as thymol, sabinene, and α-pinene were also present. Statistical analysis (principal component analysis and clustering) revealed a high variability in essential oil composition between the different organs and also within the studied periods, as the thymol proportion was higher during flowering and fruiting months. In addition, the stem, flower, and fruit essential oils were more concentrated in thymol than the leaf essential oils. The proportions of (*E*)-β-caryophyllene and α-humulene were strictly inverted with the thymol proportion throughout the harvest period or vegetative cycle. The antioxidant, anti-inflammatory and insecticidal activities of leaves and flowers essential oils were also studied. Results showed that *L. camara* leaf and flower essential oils displayed high antioxidant, anti-inflammatory and insecticidal activities.

## 1. Introduction

*Lantana camara* Lin (*L*. *camara*, Verbenaceae) is an invasive weed present in many countries [1,2,3]. This thorny shrub grows from 2 to 5 m high; its ripe fruits are blackish and its flowers are found in a variety of colors, with its flowering and fruiting seasons occurring almost year-round [4]. *L. camara* is commonly used as an ornamental plant [5,6], but also in traditional medicine in many countries [7]. Indeed, its leaves are used in infusions or decoctions to treat sores, measles, intestinal worms, fever [8], ulcer, malaria, and rheumatism [5,9]. The powdered root is used for stomach ache [8], while lotion made from the roots is used in the treatment of toothache and ulcers [10]. Whole plant infusions are also used to treat bronchitis [8]. Different solvent extracts produced from *L. camara* have already been studied, showing the occurrence of flavonoids, triterpenes, and glycosides [5]. These extracts and compounds were described as having interesting antioxidant, anti-inflammatory [11,12,13], antibacterial, cytotoxic, antifungal, anthelmintic [14,15,16,17,18], and insecticidal properties [19,20]. Some studies have also focused on *L. camara* essential oils, showing the high diversity in essential oil compositions depending on plant growth area. The major compounds reported were sesquiterpenes, such as (*E*)-β-caryophyllene, α-humulene, davanone, and germacrene D [15,21,22,23]. Some monoterpenes were also found in high proportions, such as thymol, sabinene, and limonene [24,25,26].

In a previous paper, we highlighted that essential oils produced from Ivorian *L. camara* constitute a new chemotype with high thymol contents, in comparison with essential oils hydrodistillated from *L. camara* growing in other countries [24]. However, although *L. camara* essential oils are widely used in traditional medicine, there is no data available on the impact of the variability due to phenological stage on essential oil compositions. As differences in essential oil compositions will impact their biological activities, and thus their efficiency in the treatment of target diseases, the aim of the present work was to rigorously study the variability in essential oil compositions within different *L. camara* organs, and to examine the effects of seasonal variations on essential oil compositions and their biological activities. Those were investigated monthly over a two-year period with *L. camara* growing at the same localization.

## 2. Results and Discussion

*L. camara* leaves (L), flowers (Fl), fruits (Fr), and stems (S) growing at the same harvesting place were hydrodistillated monthly over two years (June 2015–June 2017), split into two harvesting periods (June 2015–June 2016 and July 2016–June 2017).

### 2.1. Essential Oil Yield and Composition

**Figure 1:**
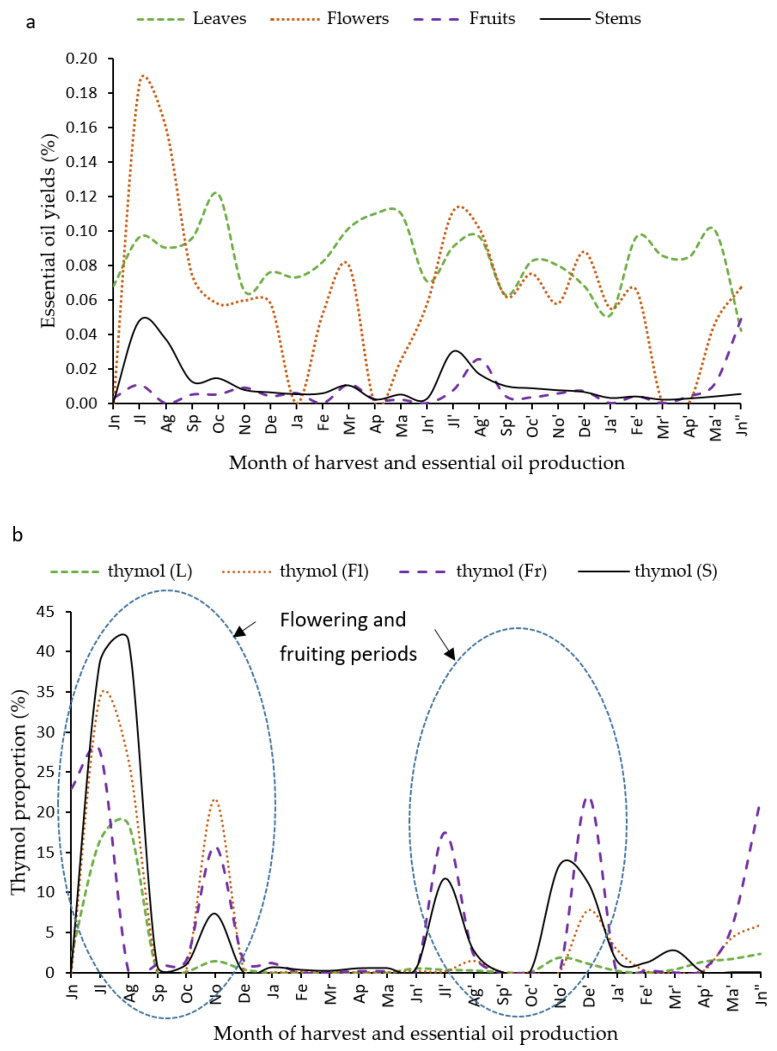
(**a**) Essential oil yield of *L. camara* organs from each month during two harvest periods. (**b**) Variation of thymol proportion in essential oil of *L. camara* organs from each month.

The hydrodistillation of *L. camara* organs produced pale yellow essential oils, whose yields fluctuated during harvest periods, as shown in Figure 1a. This high variability might arise from climatic and seasonal parameters. It should be noted that, at a given season, *L. camara* harvesting led to very low quantities of plant materials, which did not allow an accurate quantification of the essential oil yields (e.g., flowers in January of the first harvest period). Leaf essential oil yields obtained in this study (0.04–0.12%, *v*/*w*) were lower than those of *L. camara* leaves from Egypt (0.36% *v*/*w*) [27] but were close to the yield of *L. camara* fresh leaves from Brazil (0.1% *v*/*w*) [28]. Differences can result from the hydrodistillation and plant storage conditions as well as from environmental and edaphic constraints and from genetic differences between the different populations.

Table 1, Table 2, Table 3 and Table 4 report the major constituents in essential oils produced during the two-year period and their proportions. Sesquiterpene hydrocarbons were the dominant compounds in most samples but some molecules showed strong seasonal variations, including thymol, an oxygenated monoterpene. This compound was not detected in some essential oils but was the major molecule in other samples (Figure 1b).

Leaf (L) essential oils: Up to 99.1% of the total components were identified in the leaf essential oils. In all samples, (*E)-*β-caryophyllene (24.4–39.9%) and α-humulene (10.1–20.5%) were the two major molecules. Monoterpenes, such as α-pinene and sabinene, were present in lower proportions, but still reached 6.5% and 10.9%, respectively, in June 2016. Furthermore, the thymol proportion fluctuated greatly as it dominated in July (16.5%) and August (18.4%) 2015, but was lower than 2.3% for the other months (Table 1). This seasonal effect indicates an environmental influence on *L. camara* essential oil composition. Furthermore, it has been observed that the chemical composition of leaf essential oils shows high variations from one country to another and sometimes even within different regions (Table 5) [25,29,30,31,32,33,34]. Indeed, as reported herein, (*E*)-β-caryophyllene was identified here as the major compound, which was also shown in previous studies undertaken in Egypt (42.63%) [35], Algeria (26.3–47.1%) [20,36], Bangladesh (13.57%) [37], and India (23.3%) [9]. Nevertheless, two studies from Brazil highlighted bicyclogermacrene (19.42%) and isocaryophyllene (16.70%) [38], and germacrene D (24.90%) and (*E*)-β-caryophyllene (14.31%) [32] as major components, respectively (Table 5).

Flower (Fl) essential oils: Most essential oils isolated from *L. camara* flowers were dominated by (*E*)-β-caryophyllene (19.2–36.6%) and α-humulene (8.5–19.9%). However, the thymol proportion reached 34.3%, 26.3%, and 21.6% in July, August, and November 2015, respectively (Table 2), but was under 1% during other periods of the year. Sabinene and linalool (monoterpenes) were found in appreciable proportions (up to ~6%) in more than one month. Previous studies revealed the predominance of sesquiterpenes [10], with major compounds being β-elemene (14.5%) [39], (*E*)-β-caryophyllene (26.9%) in India [23], and *ar*-curcumene (27.1%) in Cameroon [34]. No *ar*-curcumene was detected in Ivorian flower essential oils but some samples were characterized by high thymol percentages, ranging from 21.6% to 34.3%. Such high levels have been reported elsewhere [24], where (*E*)-β-caryophyllene (25.9–36.6%) and α-humulene (9.9–19.9%) were also the major constituents.

**Table 5:** Major compounds of *L. camara* leaf essential oils from some countries.

Habitat	Major Compound(s)/Chemotype	Composition (%)	References
North India (Dehra Dun)	(*E*)-β-caryophyllene	23.3	[9]
	α-humulene	11.5	
	germacrene D	10.9	
	davanone	7.3	
Algeria	(*E*)-β-caryophyllene	26.3–47.1	[20,36]
	caryophyllene oxide	9.4–18.8	
	α-acoradiene	7.5–15.3	
Brazil	germacrene D	19.8	[40]
	(*E)-*β-caryophyllene	19.7	
	bicyclogermacrene	11.7	
	α-humulene	9.3	
Venezuela	germacrene D	31	[22]
	(*E*)-β-caryophyllene	14.8	
South China	germacrene D	20	[15]
	*trans*-caryophyllene	14.8	
Northeast India (Dibrugarh)	cis-davanone	47.8	[23]
	(*E*)-β-caryophyllene	10.3	
Bangladesh	(*E*)-β-caryophyllene	13.57	[37]
	α-caryophyllene	11.76	
	germacrene D	10.88	
	isocaryophyllene	9.59	
	γ-muurolene	6.85	
Benin	sabinene	38.81	[26]
	1,8-cineole	28.90	

Fruit (Fr) and Stem (S) essential oils: Table 3 and Table 4 show the predominance of sesquiterpenes in essential oils hydrodistillated from *L. camara* fruits and stems, representing 41.5–92.8% and 23.9–81.6% of their total chemical compositions, respectively. However, the fruit essential oil produced in August of the second period showed a high proportion of neral (15.3%) and geranial (23.2%). As shown in Table 3, *L. camara* stem essential oils also contained high proportions of oxygenated monoterpenes during several months: up to 41.1% of thymol and 9.2% linalool in August corresponding to the first harvesting period. In the same period, p-cymene reached 5.9% and γ-terpinene 6.2%. Palmitic acid was also present at 11.6% and 10.8% in September and October, respectively, in the second period (Table 4).

Unlike the leaves and flowers, there is less scientific data on the composition of essential oils obtained from *L. camara* fruits and stems. The composition of fruit essential oils seems to vary a lot as palmitic acid was the major constituent in south China (22.5%) [15] and Northern India (22.8%) [10], while that compound was absent from Ivorian samples from Adzope and Toumodi, which contained high proportions of (*E*)-β-caryophyllene and α-humulene [24]. Stem essential oils were dominated by germacrene D (31%) in south China [15] and by palmitic acid (32.7%) in Northern India [10]. Moreover, one fruit sample (August, second period) showed a different profile due to the high quantity of neral and geranial, which were not present in the fruit essential oils of Northern India.

### 2.2. Principal Component Analysis

#### 2.2.1. Variability of Essential Oil Composition

**Figure 2:**
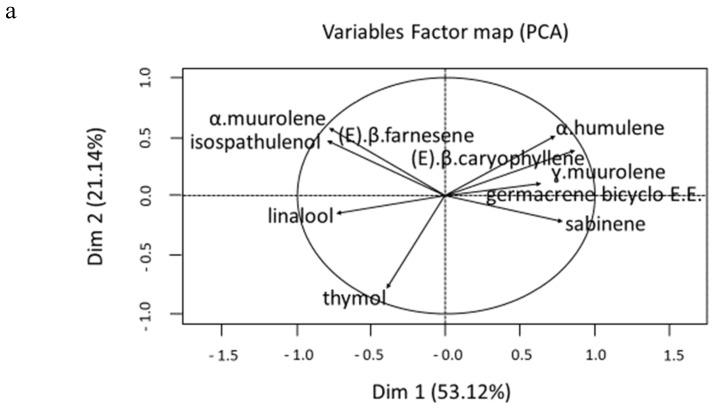

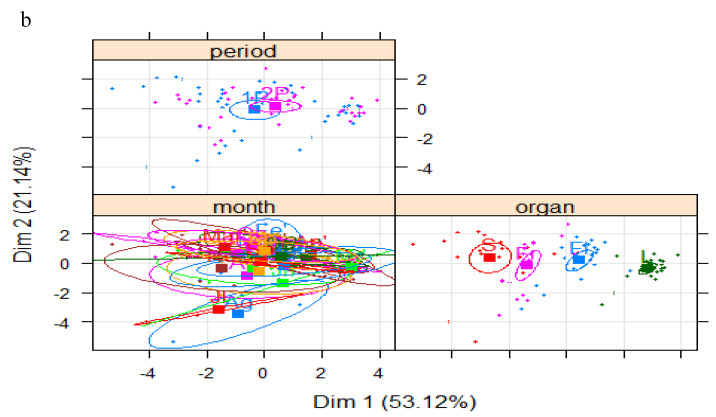
(**a**) Variables factor map of principal component analysis (PCA). (**b**) Sample map of PCA.

**Figure 3:**
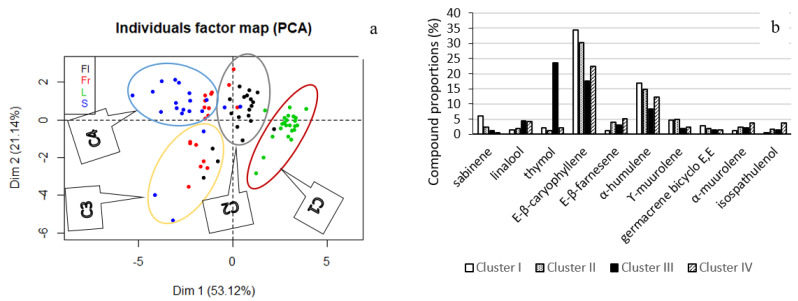
(**a**) Principal Component Analysis distribution of the essential oil samples of *L. camara* and clustering. (**b**) Proportions of the principal components of *L. camara* essential oil samples of Clusters I, II, III, and IV.

A Principal Component Analysis (PCA) was conducted to identify possible variations in the chemical composition of *L. camara* essential oils according to the month’s harvesting period. It revealed that the first and second principal components accounted for 74.26% of the total variance of the chemical composition. The first principal component (Dim1, 53.12%) was essentially composed of three opposing monoterpenes: sabinene (with positive contribution) and linalool and thymol (with negative contributions). In addition, it was noted that the second principal component (Dim2, 21.14%) was essentially built from the positive contributions of sesquiterpenes (Figure 2a). Figure 2b shows the samples map on the first principal plane which indicates that, in general, the chemical composition of essential oils from *L. camara* organs harvested at the same site is quite variable. This variability was high within the different organs, but was also present between harvesting periods and months. Indeed, for the same organ, some samples displayed very different chemical compositions. It induced, for example, the presence of essential oil samples of the same organ in different clusters that were formed by hierarchical cluster analysis (HCA). Four principal clusters can be reported (Figure 3a):

Cluster I (C1): One sample of flower essential oil and twenty-three samples of leave essential oils.

Cluster II (C2): Sixteen samples of flower essential oils, three samples of fruit essential oils, two samples of stem essential oils, and one sample of leave essential oil.

Cluster III (C3): Three samples of flower essential oils, seven samples of fruit essential oils, and three samples of stem essential oils.

Cluster IV (C4): Eight samples of fruit essential oils and fifteen samples of stem essential oils.

These four clusters were easily identified on the first principal plane of the PCA. Joint analysis of Figure 2a and Figure 3a showed that the leaf essential oil generally had a high portion of sabinene and low portion of thymol and linalool. For stem, flower, and fruit essential oils, it was the opposite, with a higher proportion of thymol and linalool and a lower presence of sabinene. The proportion and standard deviation of the principal compound of each cluster can be found in Figure 3b. Moreover, samples of clusters I, II, and IV were characterized by high proportions of (*E)-*β-caryophyllene and α-humulene, whereas cluster III samples presented a higher proportion of thymol. The compositions of the stems, flowers, and leaves differ quite clearly from each other, whereas the composition of the fruits is more variable and intermediate between that of the stems and flowers. Indeed, cluster I, which essentially consisted of leaf essential oils, could mark the stage of formation of flower buds during the vegetative cycle of the plant. Cluster II announces the formation of fruits by the abundance of flowers. Cluster III, which formed a mixture of fruit, stem, and flower samples, presented the lowest (*E)-*β-caryophyllene (17.61 ± 5.061%) and α-humulene (8.423 ± 0.948%) proportion (Figure 3b). In this cluster, the higher number of fruit samples in comparison with flowers could mark the fruiting period, hence the high production of thymol by the plant to protect itself [41].

It was noted that *L. camara* essential oil chemical compositions varied quantitatively and qualitatively from one organ to another during the plant’s life cycle. Those variations may be related to some ecological factors such as the plant’s age, the plant life cycle stage, the harvesting time, or even interindividual genetic differences between plants growing at the same location [42,43,44]. When an essential oil is produced for certain properties, a constant chemical composition is needed. The knowledge of the chemical variations during the plant’s life cycle is then crucial to select the adequate harvesting moment. This was previously reported with thymol in thyme essential oil (*Thymus vulgaris* L. ct thymol), as high compound variations were highlighted throughout the seasons, with maximal thymol yield during the flowering period [45,46]. In this study, the flowering and fruiting peaks of *L. camara* happened in July and August. It is highly possible that *L. camara* produces more thymol during the flowering and fruiting periods to protect against insect pests. Indeed, aromatic plants are molecular sources of insecticidal or insect-repelling substances capable of inducing plant protection. Some insects are particularly sensitive to the activity of oxygenated terpenes, especially phenolic ones such as carvacrol, thymol, terpineol, or linalool [41]. However, the biosynthesis of those compounds may also be induced by pathogen or pests attacks [47,48,49].

#### 2.2.2. Variability within Each Organ

The PCA was also conducted to examine more closely the interrelationships between essential oil samples of the same organ and their chemical composition according to the harvest months or periods. Cluster analysis was conducted to the study of similarity of essential oils on the basis of constituent distribution.

**Figure 4:**
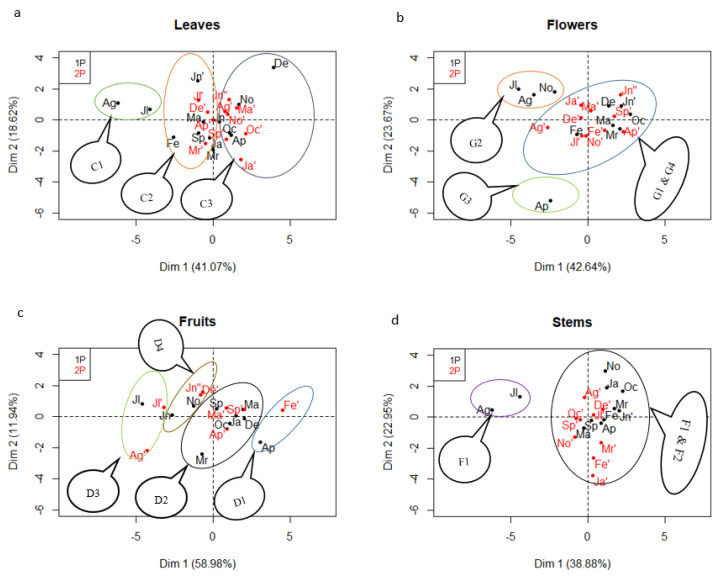
Individuals map of PCA and clustering of each organ. 1P (in black): first period, 2P (in red): second period. (**a**) C1, C2, and C3 are cluster 1, 2, and 3 of leaves, respectively. (**b**) G1, G2, G3, and G4 are cluster 1, 2, 3, and 4 of flowers, respectively. (**c**) D1, D2, D3, and D4 are cluster 1, 2, 3, and 4 of fruits, respectively. (**d**) F1, F2, and F3 are cluster 1, 2, and 3 of stems, respectively.

The cluster analysis showed three distinct parts for essential oils from leaves. July and August samples from the first harvest period displayed a very different chemical composition, with high thymol percentages, compared with the other leaf essential oils. During the second period, the chemical components of leaf essential oils were constants (Figure 4a). Moreover, it was noted that the December sample of the first period had a different chemical composition from the same month of the second period because they were not in the same cluster.

Four clusters were found for the flower essential oils. The individual factor map of the PCA (Figure 4b) shows that the chemical composition of the July, August, and November samples of the first harvest period were similar and create cluster 2 (G2). The flower essential oil sample in April of the first harvest was far away from all samples, and this sample alone constitutes the third cluster (G3). Some samples in cluster 1 (G1) and cluster 4 (G4) had chemical compositions which were not significantly different; therefore, the separation of these two groups was not clear-cut.

With regard to the chemical composition of the fruit essential oils, the PCA and clustering results revealed the formation of four distinctly separated clusters. Each cluster was constituted of samples from the first and second harvest periods. The samples from April in the first harvest and February in the second harvest presented a similarity in constituent distribution and formed the cluster 1 (D1) (Figure 4c). The presence of samples from the same month, but not the same period, were found in clusters 2 (D2), 3 (D3), and 4 (D4).

The PCA applied on the stem essential oils showed that the extracts of July and August had a similar chemical compositions compared to the other samples in the first period. The stem samples of the three months in the second harvest period (January, February, and March) showed a constant chemical composition in general (Figure 4d). The clustering suggested the occurrence of three clusters within the *L. camara* stems essential oils. Cluster 1 (F1) was clearly distinct from the other two.

### 2.3. Biological Activities

Three essential oil samples were selected on the basis of their chemical composition. These are leaf and flower essential oils from July 2015, then, leaf essential oil from July 2016.

#### 2.3.1. Insecticidal and Insect Repellent Activities

Contact insecticidal activity and insect repellent activities of the three essential oils isolated from *L. camara* were evaluated against adults of *Sitophilus granarius* (*S. granarius*). This insect is a major pest that causes dramatic grain losses during storage in silos all over the world [50,51]. Essential oils and the reference insecticide Talisma were diluted in acetone at different concentrations (6 to 42 µL/mL). Insect mortality curves were determined and LD_50_ values (Lethal Dose causing 50% of mortality after 24 h) were calculated. The LD_50_ value of the flower essential oil was 13.67 µL/mL and the other essential oils were 25.49 µL/mL and 27.38 µl/mL, respectively. The LD_50_ of Talisma was 9.87 µL/mL.

**Figure 5:**
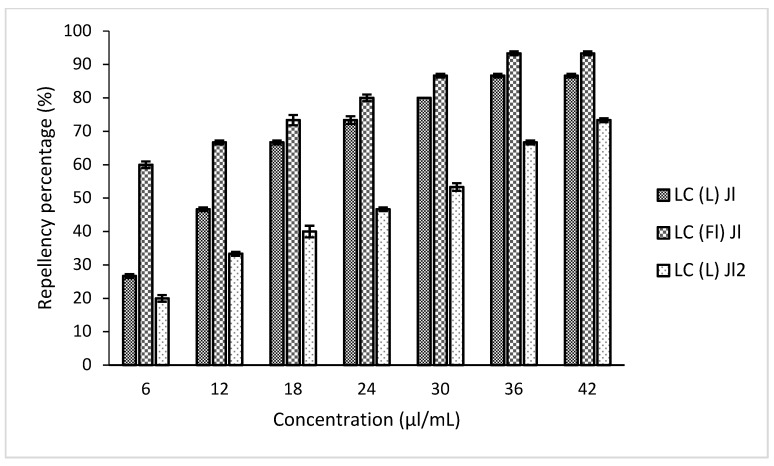
Repellency percentage of *L. camara* essential oils.

For the repellent activity evaluation, six replicates were carried out, bringing 10 adult insects into contact with two half filter paper discs, one impregnated with acetone and the other one with an essential oil solution, for each experiment. According to the classification of McDonald et al. (1970) [52], it can be said that the repellent effect of *L. camara* essential oils increases with concentration. At the concentration of 6 µL/mL, LC (L) Jl was weakly repellent (26.66 ± 0.57%), whereas LC (Fl) Jl was moderately active (60 ± 1%). However, at the maximal concentration used in the test (42 µL/mL), these two essential oil samples were more active with a repellency rate of 86.66 ± 0.57% and 93.33 ± 0.57%, respectively. In comparison, at this concentration, LC (L) Jl2 had a repellency rate of 73.33 ± 0.57% (Figure 5).

The contact toxicity test highlighted the high insecticidal potential of the flower essential oil as its LD_50_ value was close to the Talisma reference. This high insecticidal activity can be attributed to its thymol percentage (34.5%), which was lower in leaf essential oils (LC (L) Jl: 16.5%; LC (L) Jl2: 0.4%), as many studies have already showed the insecticidal power of this molecule [53,54]. Moreover, the oxygenated monoterpenes proportion in the flower essential oil (40.4%) was higher than in the leaf essential oils (19.5% and 2.9%). As monoterpenes, and especially oxygenated monoterpenes, are frequently described as insecticidal molecules [55,56,57], the highlighted activity may also be due to those molecules. Indeed, compounds such as carvacrol, linalool, eugenol, and terpineol are more toxic than camphor and α-pinene [41], and the linalool percentage was higher in the flower essential oil (2.5%) compared with the two leaf essential oils. Furthermore, essential oils are mixtures of chemical compounds of different natures and functions. Consequently, the combined action of several compounds causes a phenomena of synergy or antagonism which would be at the origin of the effectiveness of the essential oil [41,58].

Some previous studies have already focused on the insecticidal activities of essential oils and leaf extracts from *L. camara* of different origins [19,20,27,35,36,59,60]. As examples, powdered leaves of *L. camara* from the Philippines showed a high repellency for *Sitophilus zeamais* [19], while methanolic extract from India and Egyptian leaf essential oils were toxic to *Sitophilus oryzae* and *Tribolium castaneum* [35,59]. Two studies have also already demonstrated the toxicity of Algerian *L. camara* essential oils on *S. granarius*, showing that mortality increased with higher concentrations and temperatures [20] and that the essential oil becomes inefficient a long time after fumigation [36]. However, the tests used to show the toxicity in those studies were different to the present work.

#### 2.3.2. Evaluation of Essential Oil Antioxidant Activity

Essential oil antioxidant activities were evaluated by the DPPH radical scavenging assay and the ferric-reducing antioxidant power (FRAP) method, in comparison with Trolox and ascorbic acid standards.

For the DPPH radical scavenging assay, the IC_50_ was determined for each essential oil using Graph Pad Prism 8.2.1 software. This factor represents the essential oil concentration which decreased the DPPH concentration by 50%. The flower essential oil showed a greater radical scavenging activity (IC_50_: 15.53 ± 0.14 µg/mL) than those from the leaves harvested in July of the first (IC_50_: 21.96 ± 0.25 µg/mL) and second (IC_50_: 71.19 ± 1.33 µg/mL) periods. Trolox and ascorbic acid showed IC_50_ values of 12.36 ± 0.02 and 11.80 ± 0.01 µg/mL, respectively (Table 6).

**Figure 6:**
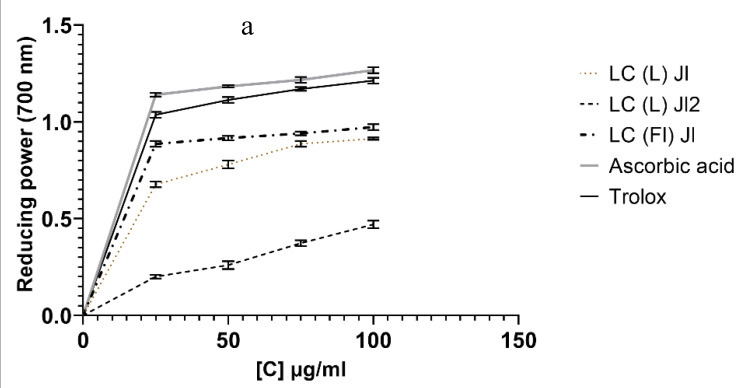

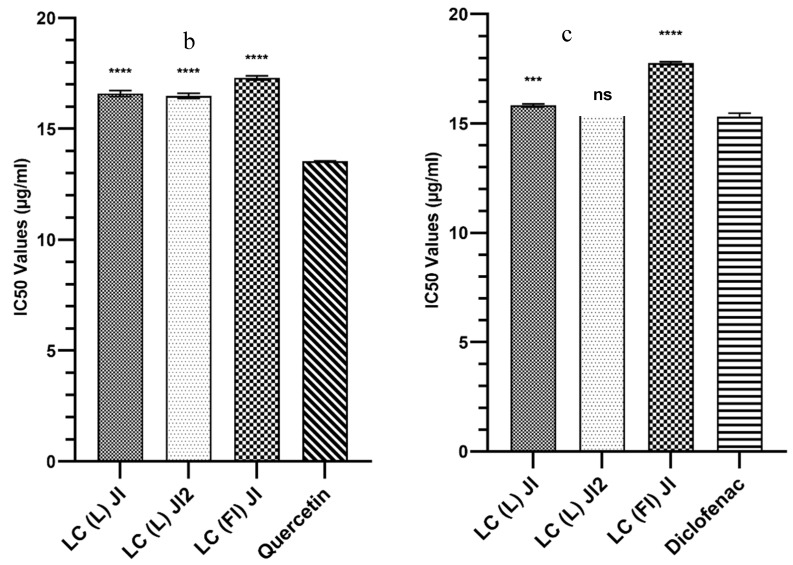
(**a**) Reducing power of *L. camara* essential oil, Trolox, and Ascorbic acid. (**b**) Anti-inflammatory by LOX inhibitory activity of *L. camara* essential oil. (**c**) Anti-inflammatory activity by denaturation of bovine albumin of *L. camara* essential oil. Values are expressed in ± SD (n = 3 experiments), ^****^ p < 0.0001, ^***^ p < 0.001, ns (non-significant), compared to Diclofenac, Quercetin (one-way ANOVA followed by Tukey’s multiple comparisons test).

For the FRAP method, results recorded in Figure 6a showed that, among the essential oil samples, the higher absorbance was observed with the flower essential oil. As higher absorbance measurements indicated a greater reducing power, this essential oil showed a high reducing power. The essential oil of the LC (L) Jl2 showed a low activity.

In the literature, the antioxidant activities of *L. camara* from diverse origins were reported [11,61,62,63,64,65,66]. However, we showed here that the composition of *L. camara* essential oils from different plant organs, and from plants growing at different places, varies widely. The results found in this study are due to thymol, whose antioxidant properties have already been demonstrated [67,68]. Moreover, the occurrence of p-cymene, borneol, β-myrcene, camphene, verbenone, 1,8-cineole, α-pinene, α-terpinene, and γ-terpinene, even in small proportions, may also contribute to the antioxidant activity [69,70,71]. This explains the lower antioxidant activity of LC (L) Jl2 essential oil, in comparison with LC (L) Jl essential oil in which 16.5% of thymol was found, but other constituents may contribute to its antioxidant activity with a probable synergistic effect.

LC (L) Jl: *L. camara* leaves essential oil of July 2015, LC (Fl) Jl: *L. camara* flowers essential oil of July 2015, LC (L) Jl2: *L. camara* leaves essential oil of July 2016

#### 2.3.3. Evaluation of Essential Oil Anti-Inflammatory Activity

The anti-inflammatory properties of *L. camara* essential oils were evaluated by the lipoxygenase (LOX) inhibition assay and by the bovine serum protein denaturation method.

In the first method, the ability of essential oils to inhibit LOX, an enzyme involved in the inflammation process, was evaluated in vitro. LOX inhibitory activity results (IC_50_) (Figure 6b) showed that all essential oil samples presented high activities. The lowest value was observed with LC (Fl) Jl which had an IC_50_ value of 17.23 ± 0.10 µg/mL. Quercetin was used as a reference (IC_50_: 13.54 ± 0.01 µg/mL). In the second method, the in vitro anti-inflammatory effect of *L. camara* essential oils was evaluated based on the denaturation of bovine albumin. The results (Figure 6c) showed that *L. camara* essential oils have an interesting IC_50_ at 15.45 ± 0.04, 15.82 ± 0.07, and 17.75 ± 0.07 µg/mL for LC (L) Jl2, LC (L) Jl, and LC (Fl) Jl, respectively. Diclofenac was used as the reference standard; its IC_50_ value was 15.31 ± 0.17 µg/mL. Additionally, the Tukey’s test showed that the difference between diclofenac and LC (L) Jl2 was not significant.

Results showed that all essential oils had interesting anti-inflammatory activities, the leaf samples displaying higher anti-inflammatory activities than the flower essential oil. The high anti-inflammatory activity of the LC (L) Jl2 sample is probably due to the high proportions of *(E)-*β-caryophyllene and α-humulene, which are particularly effective [72,73]. However, it is also possible that some other compounds interact synergistically or antagonistically. The anti-inflammatory activity of *L. camara* extracts has previously been demonstrated in several studies [12,74,75,76]. However, IC_50_ values were lower than 17.75 µg/mL in the present study (IC_50_ of *L. camara* essential oil from Peru was 81.5 µg/mL [7]), indicating that *L. camara* is a plant with anti-inflammatory properties in various forms, supporting the wide use of this plant in traditional medicine for its anti-rheumatism properties [5].

## 3. Materials and Methods

### 3.1. Plant Material

*L. camara* leaves (L), flowers (Fl), fruits (Fr), and stems (S) were collected over the two one-year periods (June 2015–June 2017) in Bregbo (5°18′23.1″N 3°49′49.6″W), 9 kilometers from Bingerville, South of Côte d’Ivoire. For harvesting, a complete experimental plan in a randomized complete block was designed in order to avoid interindividual variability. Each sample was randomly collected from 20 different plants inside the collection parcel, and plants were not collected twice during the experiment to avoid injury effects. After harvesting, the plant material was authenticated at the Centre National de Floristique (CNF), Abidjan, Côte d’Ivoire (Voucher specimen N° UCJ017433).

### 3.2. Essential Oil Hydrodistillation

Fresh plant material (0.2–1 kg) was subjected to hydrodistillation over 4 h using a Clevenger-type apparatus (solid to liquid ratio, 1:3 g:mL). Afterwards, the obtained essential oils were dehydrated with anhydrous sodium sulfate and stored in amber vials at 4 °C before further analysis.

### 3.3. Essential Oils Characterization

Essential oils hydrodistillated from the different organs of *L. camara* growing in Bregbo were analyzed using gas chromatography equipped with a flame ionization detector (GC-FID), gas chromatography-mass spectrometry (GC-MS), and the ^13^C NMR method, as described by Boué et al. (2018) and Kambiré et al. (2019) [77,78].

GC-FID analyses were carried out using a Clarus 500 Perkin Elmer (Perkin Elmer, Courtaboeuf, France) system equipped with a FID detector and two fused-silica capillary columns (50 m × 0.22 mm, film thickness 0.25 µm), BP-1 (polydimethylsiloxane), and BP-20 (polyethylene glycol). The oven temperature was programmed from 60 °C to 220 °C with an increase of 2 °C/min and then held isothermally at 220 °C for 20 min; injector temperature: 250 °C; detector temperature: 250 °C; carrier gas: helium (0.8 mL/min); split: 1/60. The relative proportions of the essential oil constituents were expressed as a percentage obtained by peak area normalization, without using correction factors. Retention index (RI) was determined by comparison with retention times of a series of n-alkanes with linear interpolation (Target Compounds software from Perkin Elmer). For GC-MS analysis, samples were analyzed with a Clarus SQ8S Perkin Elmer TurboMass detector (quadrupole), directly coupled to a Clarus 580 Perkin-Elmer Autosystem XL, equipped with a Rtx-1 (polydimethylsiloxane) fused-silica capillary column (60 m 9 0.22 mm i.d., film thickness 0.25 µm). The oven temperature was programmed to rise from 60 °C to 230 °C at 2 °C/min and then held isothermally at 230 °C for 45 min; injector temperature: 250 °C; ion-source temperature: 150 °C; carrier gas: helium (1 mL/min); split ratio: 1:80; injection volume: 0.2 mL; ionization energy: 70 eV. The electron ionization mass spectra were acquired over the mass range 35 to 350 Da.

For the NMR analysis, compounds were identified in the complex essential oil mixtures by comparison of NMR signals, chemical shifts and peak intensities with spectral data of reference compounds, compiled in a laboratory-built library using homemade software, as previously described [79,80]. In the investigated samples, the individual components were identified by NMR at proportions as low as 0.3–0.4%.

### 3.4. Insecticidal Activities

#### 3.4.1. Insect Cultures

Approximately 300 *Sitophilus granarius* (*S. granarius*) insects, the wheat grain weevil, were reared in 1 L glass jars containing sterilized wheat grains, without exposure to insecticides. The cultures were maintained in the dark at 28 ± 2 °C and 70 ± 5% relative humidity. Adults of 1–7 days old were used throughout the experiments in the same conditions.

#### 3.4.2. Insecticidal Contact Toxicity

Investigations of the insecticidal activity of the *L. camara* essential oils against *S. granarius* were carried out by direct contact application [27]. Talisma UL (Cypermetrine Biosix SA, Belgium) was used as positive control. Different concentrations, from 6 to 42 µL/mL, of essential oil and Talisma were prepared in acetone. Five-hundred microliter aliquots of the essential oil solutions were applied in a tube containing 20 g of wheat grains. After evaporation of the solvent for 20 min, 12 unsexed adults were placed into the tubes and kept at 28 ± 2 °C and 70 ± 5% relative humidity for 24 h. The control received 500 µL of acetone and was treated under the same conditions. Each essential oil and Talisma solution and the control were made in six replicates. After 24 hours, the number of dead weevils was counted and the weevil’s mortality was calculated according to the following equation [81].

Weevil mortality (%) = (Number of dead weevils/Total number of weevils) × 100

LD_50_ values were determined for each essential oil samples of *L. camara*.

#### 3.4.3. Repulsive Test

The repulsive effect of *L. camara* essential oils against *S. granarius* adults was studied according to McDonald et al. (1970) [52]. A filter paper disc (8 cm diameter) was cut into two parts. One part was impregnated with the essential oil solution (100 µL) (6 to 42 µL/mL) and the other part with acetone (100 µL). After evaporation, the impregnated paper filter was fixed with adhesive tape and placed in a petri dish. Ten adults of *S. granarius* were released into the petri dish, covered and kept at 30 ± 1 °C and 70 ± 5% relative humidity. The number of insects present in each part of the filter paper was determined 2 h after exposure. This test was used for each concentration, and the whole treatment was completed in triplicates. The repellent percentage (Pr) was calculated as follows [81].

Pr = ((Nc − Nt)/(Nc + Nt)) × 100

Nc: Number of the insects found in the piece of filter paper treated with acetone.

Nt: Number of insects found in the piece of filter paper treated with essential oil solution.

### 3.5. Antioxidant Activity

#### 3.5.1. DPPH Radical Scavenging Assay

The antioxidant activity of *L. camara* essential oils was determined by DPPH radical scavenging activity. The experimental procedure was performed using the method described by Bicas et al. (2011) [82] and modified as reported by Nea et al. (2019) [68]. The absorbance was measured at 517 nm using an Ultrospec 7000 UV–Vis dual beam spectrophotometer (GE Healthcare, Chicago, IL, USA). Both Trolox and ascorbic acid were used as positive standards. The scavenging percentage of the DPPH radical was calculated as described in the following formula,

% scavenging of DPPH radical = [(A_blank_ − A_sample_)/A_blank_] × 100

where A_blank_ is the absorbance of the reaction media without the essential oil and A_sample_ is the absorbance of the test sample. These assays were performed in triplicate and the results were expressed as mean ± SD.

#### 3.5.2. Ferric-Reducing Antioxidant Power (FRAP)

The methodology described by Hseu et al. (2008) [83] with the modifications made by Lamia et al. (2019) [84] was used to determine the reducing power of the essential oils.

A 1 mL sample of the essential oil at different concentration (25–100 μg/mL, diluted in Mmethanol) was added to 3 mL of FRAP reagent (corresponding to 1 mL 0.2 M sodium phosphate buffer pH 6.6, 1 mL 1% potassium ferricyanide solution, 1 mL 10% (*v*/*v*) trichloroacetic acid TCA). After centrifugation at 3000 g for 10 min, the supernatant was recovered and mixed with 1.5 mL distilled water and 150 μL 0.1% FeCl3. The absorbance of the mixture of essential oil and FRAP at 700 nm was then measured using a UV–Vis dual-beam spectrophotometer (GE Healthcare, Chicago, IL, USA). The absorbance of ascorbic acid and Trolox were also measured using this method, for positive control. A higher absorbance indicates a greater reducing power.

### 3.6. Anti-Inflammatory Activity

#### 3.6.1. Lipoxygenase Inhibition Assay

Lipoxygenase (EC 1.13.11.12) (LOX) from *Glycine max* was determined in vitro by the spectrophotometric method described by Tanoh et al. (2019) [85] with some modifications. A mixture of sodium borate buffer (800 µL, 0.2 M, pH 9) and soybean LOX solution (Sigma-Aldrich) (35 µL, 1000 U/mL) was incubated with different concentrations of essential oil (100 µL) in a 1 mL cell at room temperature for 15 min. After incubation, the linoleic acid substrate (35 µL, 250 µM) was added to each cell to start the reaction with the formation of hydroperoxides. The absorbance was measured at 234 nm. All tests were performed in triplicate. Quercetin (Sigma-Aldrich) was used as a standard inhibitor at the same concentration as the essential oils. Inhibition of LOX was calculated using the following equation [86].

% Inhibition = (S − E)/S × 100

E: enzyme activity without inhibitor; S: enzyme activity in the presence of the sample to be tested.

IC_50_ values (µg/mL) were determined graphically for all tested essential oils and corresponded to the essential oil concentration required to decrease the LOX activity by 50%.

#### 3.6.2. Bovine Serum Protein Denaturation Method

The bovine serum protein denaturation method assay reported by Smitha et al. (2017) [87] was used to determine the in vitro anti-inflammatory activity of each essential oil sample. The methodology was adapted for this study. Initially, the samples of essential oil and the positive control (diclofenac sodium) were dissolved in methanol for the preparation of various concentrations (25, 50, 75, and 100 μg/mL). A mixture of 0.5 mL of solution, consisting of 0.45 mL bovine serum albumin (5% *w*/*v*) and 0.05 mL of essential oil concentration or diclofenac, was incubated at 37 °C for 20 min and the temperature was then increased to keep the samples at 57 °C for 3 min. After cooling, 2.5 mL of phosphate buffer pH 6.3 was added. Each concentration was analyzed in triplicate. The reading was performed at 255 nm absorbance using a UV–Vis dual-beam spectrophotometer (GE Healthcare, Chicago, IL, USA). The percentage of inhibition was calculated by the following formula [68],

% Inhibition = [(Abs C − Abs T)/Abs C] × 100

where Abs C is the absorbance of the reactional media without inhibitor or essential oils and Abs T is the absorbance of the test sample.

### 3.7. Statistical Analysis

Mean data values are presented with standard error of the mean (mean ± SD). The data of each experiment were statistically analyzed using Graph Pad Prism 8.2.1 software (Graph Pad Software Inc., San Diego, CA, USA) followed by comparison of means (One–way ANOVA) using Tukey’s multiple comparisons test at the significance level of *p* < 0.05.

The lethal dose for 50% of the insect population (LD_50_) was calculated by the probit method of Finney (1971), for comparison of the toxicity of the essential oils tested. The percentages of mortality were transformed into probits, the regression of the logarithm of the dose versus the probits of mortality using XLSTAT-Pro 7.5 statistical software allowed the determination of the LD_50_ [88].

The multivariate analysis of the essential oil samples from *L. camara* was carried out by submitting the quantitative data of chromatographic analysis to the PCA (principal component analysis). The proportion of components in the essential oils from *L. camara* was used to define an m × n matrix, where m is the samples and n is the compounds in the essential oil. The cumulated data corresponded to the samples collected in Bregbo and consisted of 82 individuals (82 samples) and 84 variables (84 identified compounds). Among the 84 compounds in the table of data, only those containing less than 40 zero values and having a percentage of the total quantity ≥ 0.02 were selected for the PCA. The PCA and hierarchical cluster analysis (HCA) were performed by “R 3.6.0 software”.

All PCA analysis were carried out using the FactoMineR software (version 1.41), and HCA was performed using Factoextra software (version 1.0.5).

## 4. Conclusions

*L. camara* is a plant widely used in traditional medicine for the treatment of numerous afflictions. In a previous study, we already characterized a new chemotype for Ivorian *L. camara* essential oils, with high thymol contents, in comparison with essential oils obtained from *L. camara* growing in other countries. In this study, we aimed to better investigate molecular variations during the plant life cycle by studying the impact of the phenological variability on essential oil compositions over a two-year period. Statistical analysis highlighted a high chemical variability between essential oils hydrodistillated from different plant organs, but also within the same organ over the collection period, showing the effect of seasonal variability on essential oil compositions. More specifically, it was demonstrated that some compounds, such as thymol, were present in higher proportions during the flowering and fruiting months. However, despite this variability, Ivorian essential oil compositions were still different to those obtained in other countries, showing the presence of one chemotype with significant phenological variations during the plant life cycle. The high thymol proportion was correlated with a better insecticidal activity against *S. granarius*. Additionally, results showed that essential oils produced here have high antioxidant and anti-inflammatory properties, which confirms the use of this plant for efficient medical care in traditional medicine. However, large differences in biological activity were shown for essential oils hydrodistillated from different organs and for essential oils from the same organ but not the same collection month. It is well known that choosing the right chemotype and organ is crucial to use essential oils. However, our study demonstrates that the phenological stage also influences essential oil compositions. It is thus primordial to collect plants at the proper moment, correlating with peaks in active molecules, for efficient use of essential oils in traditional medicine or as an insecticide.

## Figures and Tables

**Table 1:** Major compounds and total identified compounds in leaves’ essential oils of *Lantana camara* from Bregbo.

				First Period (June 2015–June 2016)													Second Period (July 2016–June 2017)										
N°	Compounds	RIa	RIp	Jn	Jl	Ag	Sp	Oc	No	De	Ja	Fe	Mr	Ap	Ma	Jn′	Jl′	Ag′	Sp′	Oc′	No′	De′	Ja′	Mr′	Ap′	Ma′	Jn″
1	α-pinene	929	1 014	2.4	1.7	2.8	2.3	2.1	1.9	1.3	3.1	3.3	3.6	2.5	4.8	6.5	5.5	2.3	2.2	1.1	2.9	4.9	1.2	3.9	4.0	3.1	3.8
2	sabinene	964	1 122	4.8	3.0	5.2	4.7	4.8	4.6	3.2	7.5	6.3	6.9	4.9	8.8	10.9	9.5	5.1	4.8	3.2	6.1	9.0	2.5	7.3	7.6	6.2	7.7
3	linalool	1 082	1 545	1.5	1.4	1.2	1.3	1.2	1.8	2.2	1.2	1.4	1.0	1.4	1.5	2.0	1.6	1.3	1.0	1.4	1.4	1.5	0.8	1.1	1.4	1.5	1.5
4	thymol	1 267	2 176	2.1	16.5	18.4	tr	0.1	1.4	0.4	-	-	tr	tr	-	0.5	0.4	0.3	0.1	0.1	1.9	1.1	0.2	0.4	1.4	1.7	2.3
5	*E-*β-caryophyllene	1 416	1 592	35.6	29.2	24.4	33.9	35.4	35.2	33.7	34.8	31.8	36.3	37.9	35.6	32.1	32.6	34.2	36.0	39.9	34.3	33.3	37.0	35.1	34.3	35.9	35.3
6	*E-*β-farnesene	1 446	1 664	1.2	0.9	0.6	1.0	1.2	1.5	2.3	1.2	0.9	1.2	1.3	1.2	1.3	1.2	1.3	1.0	1.2	1.4	1.3	1.4	1.1	1.2	1.7	1.5
7	α-humulene	1 448	1 664	18.2	13.4	10.1	16.1	18.0	17.4	17.2	17.8	18.7	19.8	20.1	17.4	13.3	14.0	14.3	16.6	16.4	17.2	16.3	20.5	19.2	17.6	17.5	15.0
8	γ-muurolene	1 468	1 682	4.0	3.5	3.7	4.6	5.7	5.6	5.4	5.5	2.3	4.4	4.0	3.7	4.1	4.2	5.2	5.8	7.4	5.0	4.9	6.2	4.1	4.4	4.5	5.0
9	germacrene bicyclo (*E*,*E*)	1 488	1 721	3.1	2.8	1.8	2.3	3.7	3.2	2.1	0.9	0.6	2.5	2.7	3.1	3.7	3.2	3.4	4.1	5.3	2.8	2.9	2.5	2.0	3.8	3.4	5.1
10	α-muurolene	1 499	1 712	1.0	0.8	0.7	1.0	1.2	1.3	1.7	1.2	0.9	1.0	1.2	1.1	1.2	1.2	1.4	1.3	1.2	1.4	1.2	1.4	1.1	1.1	1.5	1.4
11	isospathulenol	1 615	2 217	1.1	0.3	0.3	0.5	0.8	0.9	1.4	0.4	0.3	0.6	0.8	0.3	0.3	0.4	0.8	0.7	0.5	0.8	0.4	0.5	0.5	0.7	0.6	0.6
Hydrocarbons monoterpenes (%)				11.2	9.9	18.5	10.7	10.6	10.5	7.2	14.8	14.2	15.8	11.4	20.1	25.5	22.4	11.8	11.0	6.8	13.9	20.2	6.4	17.2	17.3	14.0	17.2
Oxygenated monoterpenes (%)				4.4	19.5	21.0	2.1	1.9	3.9	3.6	2.1	3.2	1.5	2.2	2.2	3.3	2.9	4.9	1.6	1.8	3.9	3.3	1.7	2.3	3.4	4.0	4.3
Hydrocarbons sesquiterpenes (%)				75.5	61.6	51.3	73.9	79.2	77.0	76.5	75.4	68.3	77.1	79.3	73.0	66.1	67.6	72.2	79.1	85.0	74.7	71.4	84.3	74.6	73.7	76.1	73.8
Oxygenated sesquiterpenes (%)				5.1	4.5	4.1	8.8	5.0	4.7	7.5	2.8	10.5	3.1	4.6	2.0	2.0	2.6	4.5	4.9	4.6	4.0	2.1	3.8	2.9	3.2	2.7	2.2
Diterpenes (%)				0.4	0.0	0.5	0.6	0.0	0.6	1.1	0.5	0.2	0.3	0.4	0.2	0.3	0.2	0.6	0.7	0.5	0.7	0.0	0.5	0.3	0.5	0.1	0.2
Others (%)				0.0	0.2	0.2	0.1	0.0	0.1	0.2	0.0	0.3	0.1	0.2	0.4	0.8	0.7	0.1	0.2	0.4	0.1	0.3	0.2	0.2	0.1	0.3	0.2
Total identified compounds (%)				96.5	95.6	95.7	96.2	96.7	96.8	96.1	95.7	96.7	97.9	98.0	97.9	98.0	96.4	94.0	97.5	99.1	97.3	97.2	96.8	97.6	98.3	97.2	97.8

Order of elution and percentages are given on apolar column (BP-1), RIa and RIp: retention indices measured on apolar (BP-1) and polar (BP-20) columns, respectively. Identification: 1 (Kovats retention index). 2 (Mass spectrum). 3 (Nuclear Magnetic Resonance spectrum); tr = traces; Month: Ja (January); Fe (February); Mr (March); Ap (April); Ma (May); Jn (June); Jl (July); Ag (August); Sp (September); Oc (October); No (November); De (December).

**Table 2:** Major compounds and total identified compounds in flowers’ essential oils of *Lantana camara* from Bregbo.

				First Period (June 2015–June 2016)										Second Period (July 2016–June 2017)									
N°	Compounds	RIa	RIp	Jl	Ag	Oc	No	De	Fe	Mr	Ap	Ma	Jn′	Jl′	Ag′	Sp′	No′	De′	Ja′	Fe′	Ap′	Ma′	Jn″
1	α-pinene	929	1.014	0.3	0.4	0.5	0.5	1.0	1.4	0.5	2.7	1.6	0.4	1.4	1.9	0.6	1.3	2.6	0.1	0.9	0.7	1.7	0.9
2	sabinene	964	1.122	0.2	0.8	1.1	1.2	2.9	2.6	1.6	5.4	3.4	1.9	3.9	5.3	2.7	3.7	5.8	0.3	2.7	1.8	3.8	2.9
3	linalool	1.082	1.545	2.5	3.1	1.1	2.4	3.0	2.5	1.5	1.5	1.7	1.7	2.5	3.1	2.3	2.8	2.4	0.8	1.7	0.9	2.4	1.7
4	thymol	1.267	2.176	34.3	26.3	0.3	21.6	0.4	0.1	tr	tr	tr	0.2	0.1	1.4	-	0.1	7.7	2.8	0.2	0.1	4.3	5.9
5	*E-*β-caryophyllene	1.416	1.592	19.2	22.3	34.4	22.4	30.0	29.5	35.7	35.6	32.8	30.7	31.6	23.2	32.1	31.9	25.9	24.5	32.5	36.6	27.2	29.3
6	*E-*β-farnesene	1.446	1.664	2.9	2.8	5.4	3.6	4.5	4.2	4.8	1.1	4.3	4.5	3.3	2.6	4.3	3.7	4.3	2.5	3.9	4.1	4.1	4.1
7	α-humulene	1.448	1.664	8.5	10.0	16.1	10.4	14.4	15.2	17.3	19.9	15.2	14.5	14.0	9.9	15.4	15.5	11.9	11.3	16.1	16.8	12.2	12.5
8	γ-muurolene	1.468	1.682	2.6	4.4	5.3	3.6	4.9	3.2	5.1	2.0	5.2	6.2	4.3	4.2	5.3	5.0	4.3	7.7	5.0	5.3	4.9	6.8
9	germacrene bicyclo (*E*,*E*)	1.488	1.721	1.0	1.2	1.6	1.2	1.4	0.5	1.9	0.9	2.3	2.7	1.7	1.9	2.1	1.4	1.9	1.9	2.2	2.4	2.3	3.8
10	α-muurolene	1.499	1.712	1.8	1.7	2.8	2.0	2.6	2.1	2.6	1.1	2.5	2.6	2.1	1.6	2.8	2.1	2.3	1.6	2.2	2.5	2.3	2.5
11	isospathulenol	1.615	2.217	0.7	0.9	2.0	1.7	2.8	1.4	1.4	0.3	1.6	1.9	1.2	0.8	1.8	1.2	1.4	1.2	1.5	1.5	1.6	2.0
Hydrocarbons monoterpenes (%)				5.1	3.8	3.1	5.9	7.3	7.5	4.6	11.8	9.1	5.0	11.0	29.8	6.7	9.6	15.9	1.3	7.5	5.5	10.5	8.1
Oxygenated monoterpenes (%)				40.4	32.4	1.8	28.2	5.1	10.3	2.5	2.3	2.5	2.7	3.8	7.1	3.2	4.4	11.4	4.0	2.5	1.5	12.2	8.3
Hydrocarbons sesquiterpenes (%)				46.5	55.4	82.7	55.2	73.4	67.8	81.8	71.0	76.2	77.1	71.7	53.7	77.2	74.2	63.3	66.4	76.8	81.8	64.9	71.7
Oxygenated sesquiterpenes (%)				3.1	4.7	9.0	5.4	10.5	9.7	6.3	11.1	7.2	8.3	8.1	3.2	8.2	6.5	4.7	16.0	6.7	6.1	5.1	4.9
Diterpenes (%)				0.0	0.0	0.2	0.1	0.3	0.2	0.3	0.4	0.3	0.4	0.3	0.2	0.4	0.3	0.2	0.3	0.3	0.3	0.2	0.3
Others (%)				0.1	0.3	0.1	0.5	0.1	0.2	0.3	0.1	0.1	0.6	0.1	0.1	0.0	0.3	0.1	0.0	0.1	0.0	0.6	0.1
Total identified compounds (%)				95.2	96.6	96.9	95.2	96.7	95.7	95.8	96.6	95.4	94.1	95.1	94.1	95.7	95.2	95.6	88.0	93.8	95.2	93.4	93.4

Order of elution and percentages are given on apolar column (BP-1), RIa and RIp: retention indices measured on apolar (BP-1) and polar (BP-20) columns, respectively. Identification. 1 (Kovats retention index). 2 (Mass spectrum). 3 (Nuclear Magnetic Resonance spectrum); tr = traces; Month: Ja (January); Fe (February); Mr (March); Ap (April); Ma (May); Jn (June); Jl (July); Ag (August); Sp (September); Oc (October); No (November); De (December).

**Table 3:** Major compounds and total identified compounds in fruits’ essential oils of *Lantana camara* from Bregbo.

				First Period (June 2015–June 2016)										Second Period (July 2016–June 2017)							
N°	Compounds	RIa	RIp	Jn	Jl	Sp	Oc	No	De	Ja	Mr	Ap	Ma	Jl′	Ag′	Sp′	De′	Fe′	Ap′	Ma′	Jn″
1	sabinene	964	1.122	0.4	3.0	1.1	-	0.7	0.2	0.4	1.6	-	0.1	3.9	2.4	0.8	0.6	0.4	1.6	2.6	0.7
2	linalool	1.082	1.545	3.8	6.0	4.8	3.5	4.9	3.6	4.0	4.2	0.7	5.5	5.8	3.9	4.3	4.5	2.0	2.7	4.3	5.0
3	neral	1.213	1.675	-	0.2	0.1	-	1.0	0.4	0.8	-	-	0.1	0.2	15.3	0.2	0.1	-	0.1		0.1
4	geranial (citral)	1.241	1.730		-	-	0.5	1.3	-	1.3	-	-	-	-	23.2	-	-	-	-		-
5	thymol	1.268	2.180	23.0	27.6	1.0	1.5	15.8	1.6	1.2	tr	0.2	0.1	17.5	2.3	-	22.1	0.3	0.4	5.6	21.5
6	*E-*β-caryophyllene	1.416	1.592	20.5	15.1	27.1	23.7	21.4	27.0	29.7	24.8	29.5	29.4	18.7	11.2	28.3	20.8	36.9	29.2	26.5	19.4
7	*E-*β-farnesene	1.446	1.664	2.9	2.6	4.0	4.5	4.1	5.3	5.1	3.6	5.2	5.0	3.1	1.8	5.0	4.5	6.3	3.4	5.3	4.6
8	α-humulene	1.448	1.664	10.5	7.2	13.1	13.6	10.6	14.1	15.1	14.7	17.3	16.7	8.8	5.6	13.9	10.3	18.3	16.0	12.8	9.2
9	γ-muurolene	1.468	1.682	0.7	0.5	1.0	2.7	0.7	3.5	1.1	1.3	4.5	3.5	2.4	1.5	4.0	2.5	4.8	3.7	3.5	3.5
10	germacrène bicyclo (*E*.*E*)	1.489	1.721	1.7	1.2	2.1	1.4	1.5	1.4	1.7	tr	1.4	1.7	1.4	0.5	1.9	1.9	2.5	2.0	1.7	1.7
11	α-muurolene	1.499	1.712	2.0	1.7	2.8	2.8	2.5	3.2	3.2	2.9	3.6	3.3	2.0	1.3	3.5	2.6	3.8	2.5	3.2	2.8
12	isospathulenol	1.616	2.222		1.3	2.4	3.0	2.2	3.2	2.6	1.8	1.7	2.4	1.5	1.4	2.7	1.8	2.3	1.7	2.5	2.3
Hydrocarbons monoterpenes (%)				2.0	15.3	4.9	0.0	5.1	1.4	2.3	4.4	0.5	1.4	15.5	7.6	3.2	3.8	1.6	6.8	6.4	3.4
Oxygenated monoterpenes (%)				30.7	37.4	8.4	7.0	26.5	7.7	8.4	5.7	1.4	8.2	26.1	52.6	6.3	29.2	2.9	5.4	16.5	29.3
Hydrocarbons sesquiterpenes (%)				50.5	37.3	67.8	63.0	53.8	69.2	73.4	60.9	75.0	73.1	45.4	28.3	72.1	53.3	86.1	70.6	64.9	52.4
Oxygenated sesquiterpenes (%)				6.8	4.2	9.2	13.4	7.1	13.0	9.8	21.4	13.1	10.8	5.0	4.7	10.1	6.0	6.7	8.8	6.6	6.9
Diterpenes (%)				0.0	0.5	1.8	0.6	0.1	2.1	1.7	1.1	2.8	1.3	0.7	0.4	1.8	1.0	1.2	0.8	0.8	1.5
Others (%)				0.7	0.7	2.2	0.7	2.0	0.9	0.9	0.8	2.5	0.9	1.2	1.2	0.7	2.5	0.2	0.8	0.9	0.5
Total identified compounds (%)				90.6	95.3	94.3	84.7	94.7	94.3	96.6	94.3	95.3	95.7	93.9	94.7	94.3	95.7	98.7	93.3	96.2	94.0

Order of elution and percentages are given on apolar column (BP-1), RIa and RIp: retention indices measured on apolar (BP-1) and polar (BP-20) columns, respectively. Identification. 1 (Kovats retention index). 2 (Mass spectrum). 3 (Nuclear Magnetic Resonance spectrum); tr = traces; Month: Ja (January); Fe (February); Mr (March); Ap (April); Ma (May); Jn (June); Jl (July); Ag (August); Sp (September); Oc (October); No (November); De (December).

**Table 4:** Major compounds and total identified compounds in stems’ essential oils of *Lantana camara* from Bregbo.

				First period (June 2015–June 2016)												Second period (July 2016–June 2017)								
N°	Compounds	RIa	RIp	Jl	Ag	Sp	Oc	No	Ja	Fe	Mr	Ap	Ma	Jn′	Jl′		Ag′	Sp′	Oc′	No′	De′	Ja′	Fe′	Mr′
1	sabinene	964	1.122	0.2	0.9	2.6	0.1	0.3	1.0	0.4	-	-	-	0.6	-		0.4	-	-	-		0.4	-	-
2	p-cymene	1.011	1.270	0.9	5.9	0.4	0.1	0.3	0.3	0.4	-	-	tr	0.1	-		0.1	-	-	0.2	0.1	0.1	-	-
3	γ-terpinene	1.047	1.244	0.4	6.2	0.2	0.1	0.1		0.2	-	-	-	0.2	-		0.2	-	-	0.1	0.1	0.1	-	-
4	linalool	1.082	1.545	9.0	5.4	4.7	7.2	9.2	8.3	5.8	1.3	1.4	2.4	6.5	2.6		6.2	0.5	0.2	2.9	4.6	1.3	0.8	2.4
5	thymol	1.268	2.180	38.6	41.4	0.8	0.9	7.3	0.6	0.3	0.2	0.5	0.5	0.4	11.7		2.8	-	0.2	13.4	11.1	1.4	1.2	2.7
6	*E-*β-caryophyllene	1.416	1.592	9.9	7.3	24.6	24.0	17.0	19.1	24.8	19.1	20.2	19.4	22.5	19.6		16.2	14.2	14.0	20.7	19.0	25.3	24.5	24.8
7	*E-*β-farnesene	1.446	1.664	2.8	1.4	4.1	6.2	6.9	6.1	5.7	5.6	4.3	4.4	6.4	5.3		4.9	4.0	4.5	3.6	6.3	3.0	4.1	4.0
8	α-humulene	1.448	1.664	5.2	3.2	12.4	12.8	9.3	11.6	14.6	13.1	13.1	11.0	11.5	10.1		7.8	8.7	7.2	10.0	11.0	13.6	15.4	14.7
9	γ-muurolene	1.468	1.682	1.2	0.3	1.0	0.8	2.1	2.4	0.8	1.9	2.3	2.6	3.9	2.9		2.5	2.5	2.4	2.5	2.6	6.4	2.9	3.1
10	germacrene bicyclo (*E*,*E*)	1.489	1.721	1.2	1.1	2.4	2.7	0.9	0.5	1.9	1.3	1.3		2.6	1.1		0.9	0.7	0.7	2.7	2.7	2.0	1.4	1.2
11	α-muurolene	1.499	1.712	2.2	1.2	3.3	4.8	5.0	4.7	3.6	4.8	3.5	3.3	4.8	4.3		3.8	3.8	3.5	3.0	4.6	2.5	2.8	3.6
12	isospathulenol	1.616	2.222	2.4	1.3	3.4	6.0	6.2	5.2	2.6	5.8	5.7	3.1	4.2	4.1		4.9	4.2	4.0	2.4	3.5	2.0	1.6	3.1
13	palmitic acid	1.934	2.875	0.5	-	-		1.4			1.8	2.6	4.9	1.3	1.5		2.1	11.6	10.8	1.2	0.9	1.3	4.6	1.4
14	phytol E	2.098	2.604	1.4	-	1.5	1.6	3.3	2.2	0.9	6.2	3.4	10.2	5.3	6.6		5.8	8.7	17.9	6.0	4.1	2.7	12.7	3.4
Hydrocarbons monoterpenes (%)				2.9	19.3	7.5	0.9	1.4	2.8	2.0	0.0	0.0	0.0	2.1	0.0		9.7	0.0	0.0	0.4	0.2	1.4	0.0	0.0
Oxygenated monoterpenes (%)				52.0	50.9	7.4	9.4	18.4	12.0	12.3	4.2	4.9	4.8	8.4	15.1		11.7	0.6	0.4	18.5	17.4	3.0	2.0	7.0
Hydrocarbons sesquiterpenes (%)				29.6	21.0	64.5	68.5	52.7	57.3	66.8	57.4	56.5	53.1	64.2	55.7		49.6	47.6	44.4	59.2	60.0	69.7	67.0	64.5
Oxygenated sesquiterpenes (%)				6.1	2.9	8.9	11.1	14.9	15.3	8.1	21.2	21.3	17.1	11.0	11.7		12.9	15.3	9.7	8.8	9.9	11.9	7.1	11.5
Diterpenes (%)				1.4	0.0	1.5	1.6	3.3	2.2	0.9	6.2	3.4	10.2	5.3	6.6		5.8	8.7	17.9	6.0	4.1	2.7	12.7	3.4
Others (%)				2.8	2.4	2.9	3.1	3.2	2.9	3.2	3.0	5.1	7.3	4.4	4.6		5.1	15.8	12.0	4.8	5.6	3.5	8.2	4.5
Total identified compounds (%)				94.8	96.4	92.8	94.5	93.9	92.4	93.4	92.0	91.2	92.4	95.5	93.7		94.8	88.0	84.4	97.6	97.2	92.2	96.9	90.9

Order of elution and percentages are given on apolar column (BP-1), RIa and RIp: retention indices measured on apolar (BP-1) and polar (BP-20) columns, respectively. Identification. 1 (Kovats retention index). 2 (Mass spectrum). 3 (Nuclear Magnetic Resonance spectrum); tr = traces; Month: Ja (January); Fe (February); Mr (March); Ap (April); Ma (May); Jn (June); Jl (July); Ag (August); Sp (September); Oc (October); No (November); De (December).

**Table 6:** IC_50_ values of antioxidant activity of *L. camara* essential oil by 2,2-diphenyl-1-picrylhydrazyl (DPPH) assay. Data are expressed as the mean of triplicates.

Samples	IC_50_ (µg/mL)
Trolox	12.36 ± 0.02
LC (L) Jl	21.96 ± 0.25 ****
LC (Fl) Jl	15.53 ± 0.14 ***
LC (L) Jl2	71.19 ± 1.33 ****
Ascorbic acid	11.80 ± 0.01

Tukey’s test vs. Trolox and Ascorbic acid. **** *p* < 0.0001; *** *p* < 0.001.
